# *Acinetobacter baumannii* LOS Regulate the Expression of Inflammatory Cytokine Genes and Proteins in Human Mast Cells

**DOI:** 10.3390/pathogens10030290

**Published:** 2021-03-03

**Authors:** Takane Kikuchi-Ueda, Tsuneyuki Ubagai, Go Kamoshida, Ryuichi Nakano, Akiyo Nakano, Yasuo Ono

**Affiliations:** 1 Department of Microbiology and Immunology, School of Medicine, Teikyo University, Tokyo 173-8605, Japan; ubat@med.teikyo-u.ac.jp (T.U.); onoy@med.teikyo-u.ac.jp (Y.O.); 2 Department of Microbiology and Infection Control Sciences, Division of Biological Sciences, Kyoto Pharmaceutical University, Kyoto 607-8412, Japan; kamoshida@mb.kyoto-phu.ac.jp; 3 Department of Microbiology and Infectious Diseases, Faculty of Medicine, Nara Medical University, Nara 634-8521, Japan; rnakano@naramed-u.ac.jp (R.N.); akiyo@naramed-u.ac.jp (A.N.)

**Keywords:** *Acinetobacter baumannii* LOS, mast cells, IL-8

## Abstract

Herein, we investigated the effect of bacterial lipooligosaccharides (LOS), from *Acinetobacter baumannii*, on the expression of pro-inflammatory genes that play an essential role in bacterial clearance. LAD2 human mast cells were stimulated with LOS derived from two strains of *A. baumannii*—ATCC 19606 and MDRA T14. LOS exposure induced the expression of genes for pro-inflammatory mediators, including TNF-α, IL-8, LTC4S, CCL4, and TLR4. The mRNA expression levels of a majority of the pro-inflammatory genes, except TLR4, in *A. baumannii*-LOS stimulated mast cells were increased. Moreover, co-culture of neutrophils with the supernatant obtained from LOS (ATCC 19606 and MDRA T14)-induced LAD2 cells increased the transmigration of neutrophils, which plays a critical role in the early protection against bacterial infections. The results of the present study suggest that LOS could be involved in the pathogenicity of *A. baumannii* by inducing inflammatory responses via mast cells and that IL-8 is involved in recruiting neutrophils in response to bacterial invasion.

## 1. Introduction

*Acinetobacter baumannii* is an emerging pathogen leading to hospital- and community-acquired infections worldwide. *Acinetobacter baumannii* is a Gram-negative coccobacillus bacteria, and its ability to acquire drug resistance has become a major concern [1,2]. Furthermore, the ability of *A. baumannii* to form biofilms, which are composed of bacterial pili [3], the outer membrane protein OmpA [4], the autoinducer synthase, extracellular polymeric substances (EPS) [5], and the bacteria themselves, allows it to grow in unfavorable conditions and protects it from antimicrobials [6]. Abiotic surfaces, such as mechanical ventilators or intravascular devices connected to patients with wounds or burns, are prone to *A. baumannii* infection [7,8].

Mast cells have been recognized for their roles in the initiation of various allergic diseases and helminthic infections [9]. They are found in large numbers in mucosal areas, skin, and perivascular tissues and are long-term residents of tissues. The presence of mast cells, particularly in areas in close contact with the external environment, implies their role in the early recognition of pathogens. They can modulate the host’s innate response through the secretion of mediators. For example, mast cells were reported to promote the clearance of bacteria through the release of various mediators, such as TNF-α, which is a chemoattractant for neutrophils [10,11]. Several studies have reported that bacteria-derived components affect mast cell functions [12,13]. Lipopolysaccharide (LPS) or lipooligosaccharide (LOS), a major constituent of the outer membrane of Gram-negative bacteria, interacts with mast cells. For instance, human normal cord blood-derived mast cells were activated by *Escherichia coli* LPS and released TNF-α [13,14]. Previously, we have reported that *A. baumannii* adheres to mast cells via CD32 expressed on mast cells and induces pro-inflammatory TNF-α, which results in the progression of inflammation with the recruitment of activated neutrophils [15]. However, the interaction of *A. baumannii* LOS with mast cells remains unclear. *A. baumannii,* which was used in this study, did not produce LPS as its incapable of synthesizing O-antigen and instead produced LOS (Figure S1) [16]. The aim of this study was to investigate, for the first time, whether the exposure of mast cells to *A. baumannii* components affects the expression of pro-inflammatory cytokines, chemokines, leukotriene synthase, and Toll-like receptor 4 (TLR4) during bacterial infection in vitro.

## 2. Results

### 2.1. Effect of LOS on Mast Cell Gene Expression of Pro-Inflammatory Genes

**Figure 1:**
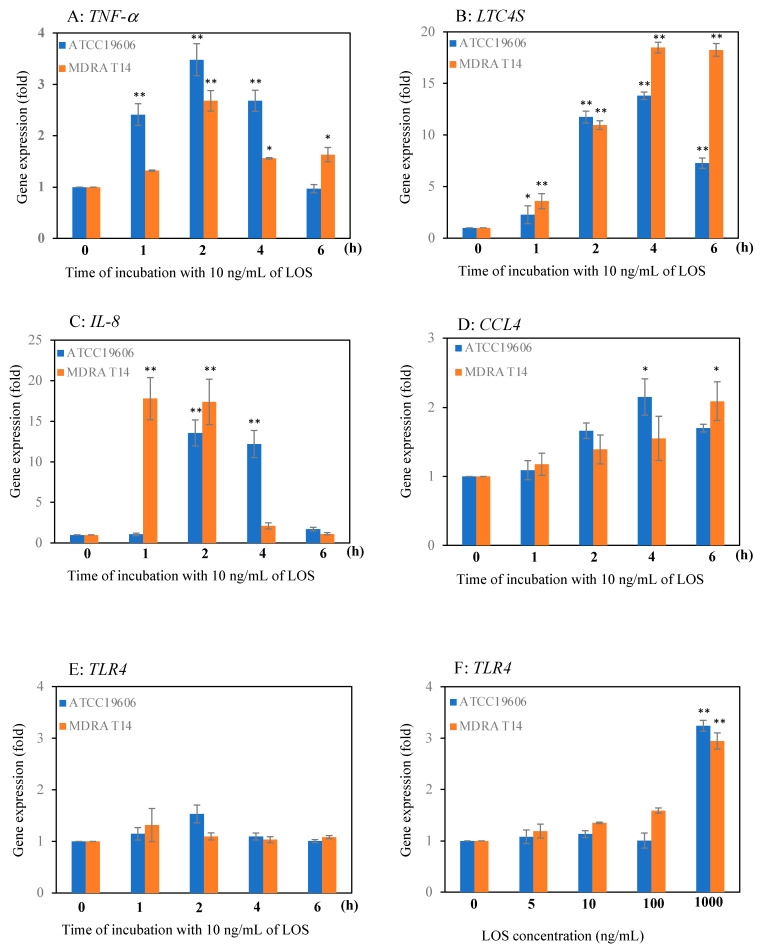
The gene expression levels in LAD2 cells stimulated with lipooligosaccharide (LOS) derived from *A. baumannii*. Both 10 ng/mL of LOS induced mRNA expression of the pro-inflammatory mediators, (**A**) *TNF-α* and (**B**) *LTC4S* and mRNA expression of the CXC chemokines. (**C**) *IL-8* and (**D**) *CCL4.* The expression levels of the target genes in LAD2 cells were estimated after incubation with LOS for 1, 2, 4, and 6 h and were normalized to β-actin gene-expression levels. Eventually, the fold changes in expression of cytokine mRNA between the LOS treated cells and time zero were presented. Expression levels of *TLR4* in LAD2 cells stimulated with LOS derived from *A. baumannii*. (**E**) The mRNA- expression compared with that for control LAD2 cells cultured in medium (0 h) after exposure with 10 ng/mL LOS (ATCC 19606, T14). (**F**) The *TLR4* mRNA-expression in cells treated with 5, 10, 100, and 1000 ng/mL of LOS compared with that for control (0 ng/mL LOS) after 4 h. * *p* < 0.05, ** *p* < 0.01 with the LAD2 control (0 h).Error bars represent the standard error. The data shown are representative of at least three independent experiments.

The effect of LOS stimulation on the expression of inflammatory mediators released from mast cells was investigated by real-time PCR. It was observed that the mRNA expression level of *TNF-α* increased by 3.5-fold and 2.6-fold in cells that were stimulated with LOS derived from *A. baumannii* ATCC 19606 and clinical isolate multi-drug resistant MDRA T14 for 2 h, respectively (Figure 1A). The expression levels of the target genes were normalized to β-actin gene-expression levels and compared to gene-expression levels of LAD2 cells at time zero. *LTC4S* gene encodes leukotriene C4 synthase that converts leukotriene A4 and glutathione to leukotriene C4. Leukotriene C4 is one of the inflammatory mediators of mast cells. The mRNA expression levels of *LTC4S* after exposure to LOS derived from MDRA T14 for 1, 2, 4, and 6 h were found to increase gradually by 3.6, 11.0, 18.5, and 18.2-fold, respectively. Similarly, it was increased 2.3, 11.7, 13.8, and 7.3-fold, respectively, after exposure to ATCC 19606 (Figure 1B). These results indicated that the highest *TNF-α* mRNA expression was observed after 2 h LOS exposure in ATCC19606 and MDRA T14, whereas the highest *LTC4S* mRNA expression was observed after 4 h of incubation.

### 2.2. Effect of LOS on Expression of Chemoattractant Genes in Mast Cells

The mRNA-expression level of *IL-8* was increased 17.4- and 13.6-fold after 2 h stimulation of cells with LOS derived from MDRA T14 and ATCC 19606, respectively (Figure 1C). In contrast, only a 2.2-fold increase in the *CCL4* mRNA expression level was noted after 4 h stimulation with LOS from *A. baumanii* 19606, and a 2.1-fold increase after exposure to LOS derived from MDRA T14 at 6 h (Figure 1D).

### 2.3. Effect of LOS on the Expression of the TLR4 Gene in Mast Cells

The expression level of TLR4 mRNA in mast cells stimulated with LOS (10 ng/mL) derived from *A. baumannii* showed no significant difference between ATCC 19606 and MDRA T14 (Figure 1E). Moreover, its expression level did not change with an increase in the dose of LOS (5 ng/mL to 100 ng/mL) (Figure 1F).

### 2.4. Effect of LOS on Mast Cell Production of Pro-Inflammatory Mediators

**Figure 2:**
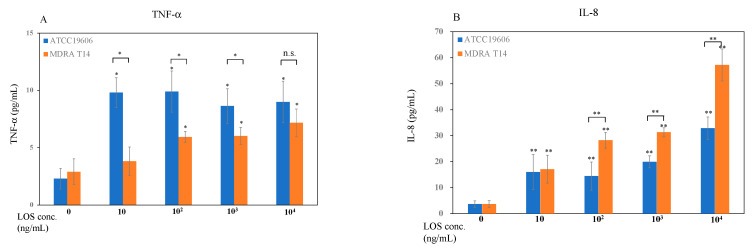
Cytokine and chemokine levels in culture supernatants of LAD2 cells stimulated with LOS derived from *A. baumannii*. (**A**) TNF-α and (**B**) IL-8. The data are shown as the mean with standard error. * *p* < 0.05, ** *p* < 0.01, compared with LAD2 control supernatant. n.s.: not significant.

We examined the effect of bacterial LOS from *A. baumanii* (19606 and MDRA T14) on LAD2 cell degranulation/production of pro-inflammatory mediators. The results revealed that bacterial LOS used in this study induced TNF-α release from mast cells in a dose-dependent manner (Figure 2A). LOS from *A. baumannii* ATCC 19606, even at the lowest dose (10 ng/mL), induced more TNF-α release as compared to LOS derived from the drug resistant strain T14. Notably, TNF-α production in cells treated with LOS derived from the drug-sensitive strain ATCC 19606 (9.8 pg/mL) was significantly higher than that in cells treated with LOS derived from the drug-resistant strain T14 (3.8 pg/mL). Bacterial LOS stimulation induced IL-8 production from mast cells in a dose-dependent manner (Figure 2B). MDRA T14 LOS at high concentrations (100 ng/mL and 1000 ng/mL) produced higher IL-8 (28.2 and 31.3 pg/mL) than did ATCC 19606 LOS (14.4 and 19.9 pg/mL), respectively. Other pro-inflammatory cytokines (IL-1β, IL-6, IL-10, and IL-12p70) were not measurable.

### 2.5. Effect of Pro-Inflammatory Mediators Released from Mast Cells Stimulated with A. baumannii LOS to Induce Neutrophil Transmigration

**Figure 3:**
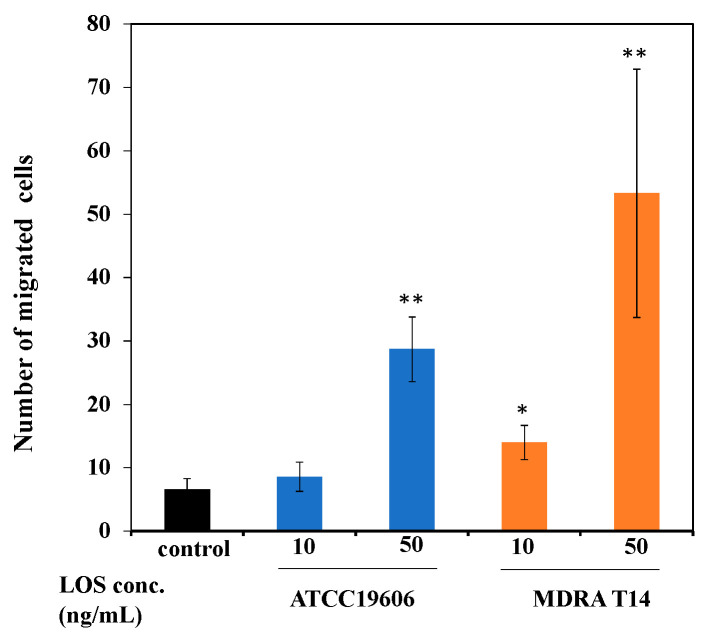
*Acinetobacter baumannii* LOS induced neutrophil transmigration across Boyden-chamber membranes. The data shown are the mean with standard errors of results for 10 randomly selected fields. The bars indicate the mean values from 10 fields. * *p* < 0.05, ** *p* < 0.01, compared with control LAD2 cells. Error bars represent the standard error. Data are representative of at least three independent experiments.

A neutrophil transmigration assay was performed to elucidate the role of the pro-inflammatory mediators released from mast cells stimulated with *A. baumannii* LOS. The results showed that the mast cells stimulated with LOS from MDRA T14 (10 or 50 ng/mL) induced more neutrophil transmigration as compared to those stimulated with LOS from ATCC 19606 (Figure 3), suggesting that LOS derived from MDRA T14 may have more inflammatory effects than LOS derived from drug-sensitive ATCC 19606.

## 3. Discussion

We studied the immune responses of human mast cells toward LOS derived from *A. baumannii* for the first time. We analyzed the expression profiles of pro-inflammatory mediators in LAD2 cells stimulated with LOS from *A. baumannii* ATCC19606 and MDRA T14 to elucidate the pathophysiology of the bacterial strains. We used 10 ng/mL LOS to stimulate LAD2 cells and investigated the successive changes in mRNA expression. It was previously reported that LPS/LOS concentrations lower than 0.5 ng/mL in blood could cause severe septic syndromes in humans [17]. TNF-α is a potent mediator found abundantly in granules of mast cells and is released by degranulation upon bacterial infection for clearing pathogens [11,15,18]. Several studies have reported the release of TNF-α in response to LPS derived from *E. coli* [13,14]. Here, we examined the *TNF- α* gene expression in mast cells stimulated with LOS derived from *A. baumannii*. *TNF-α* mRNA expression was upregulated, with the highest levels observed after a 2 h exposure to LOS from *A. baumannii* ATCC 19606, MDRA T14 (Figure 1A). These results supported the previous findings on the role of *TNF-α* in host defense against bacterial invasion.

It is known that mast cells produce leukotrienes (LTs) upon bacterial infection [19,20,21]. Malaviya et al. reported that mouse bone marrow-cultured cells (BMMC) released significant amounts of LTB4 and LTC4 in response to exposure to FimH-expressing type 1 fimbriated *E. coli* in vitro [11]. LTB4 is a potent chemotactic factor for neutrophils [19,20,22] and eosinophils [21,22,23,24], whereas LTC4 is the parent compound of the cysteinyl leukotrienes (CysLTs). Peters-Golden et al. reported that cell specificity exists in the profile of LTs generated in mast cells, while eosinophils synthesize CysLTs primarily [24]. LOS are important pathogen-associated molecular patterns (PAMPs) that signal via TLR4. Aderem et al. reported that LT synthesis from mouse macrophages was enhanced after a 60 min exposure to 1 μg/mL of *E. coli* LPS [25]; however, Coffey et al. reported that prolonged exposure of macrophages to *E. coli* LPS impaired their capacity for LT synthesis in response to activating stimuli [26]. Nevertheless, the gene involved in synthesizing LTC4 by stimulating with *A. baumannii* LOS is still unknown. Here, we examined the expression profile of *LTC4S* encoding leukotriene C4 synthase in LAD2 cells stimulated with *A. baumannii*-derived LOS. The results revealed upregulation of the *LTC4S* mRNA levels in response to the LOS derived from MDRA T14 strains (Figure 1B), suggesting that *A. baumannii* LOS-mediated effects may involve LTC4 synthesis rather than TNF-α synthesis.

MDRA T14-LOS stimulation induced quicker (1-h exposure) and higher (18-fold) *IL-8* mRNA expression than did ATCC 19606-LOS stimulation (2-h exposure, 13-fold), respectively, in LAD2 cells (Figure 1C). IL-8 levels in the culture supernatant of LAD2 cells stimulated with ATCC 19606 and MDRA T14 LOS (Figure 2B) were found to vary in a dose-dependent manner. Interestingly, the number of transmigrated neutrophils in the group stimulated with MDRA T14-LOS was higher than in the one stimulated with ATCC 19606-LOS (Figure 3). These findings were found to contradict the results obtained in our previous study that TNF-α was the primary factor for neutrophil transmigration and activation when LAD2 cells were infected with live *A*. *baumannii* [15]. The discrepancy could have occurred because the cells were stimulated by LOS in this study, which is a soluble bacterial component, while in the previous study, cells were stimulated by whole bacteria that had outer membrane proteins and adhesion factors, such as pili, in addition to LOS. Thus, it can be suggested that *TNF-α* could be responsible for the recruitment of neutrophils when additional stimulation factors other than LOS are involved, whereas IL-8 plays this role when LPS is the only stimulation factor.

CCL4 is a potent chemoattractant for leukocyte migration [20,22]. Sun et al. reported that CCL4 expression at the gene and protein levels in human mast cells was increased after *P. aeruginosa* infection [27]. We also observed that *CCL4* increased in LAD2 cells infected with *A. baumannii* (Figure S2). However, the expression levels of *CCL4* in LAD2 cells stimulated with *A. baumanni* LOS were lower than those in cells stimulated with live *A. baumannii*. These findings suggest that IL-8 released from LAD2 cells by *A. baumannii* LOS stimulation could be the major factor for neutrophil migration.

There are several reports on the responses of mast cells via TLR4 to PAMPS [13,14,28]. Wierzbicki et al. reported that mast cells collected from the peritoneal cavities of rats generated and released significant amounts of leukotriene when activated with peptidoglycan from *Staphylococcus aureus*, LPS from *E. coli*, or lipoarabinomannan from *Mycobacterium smegmatis* [20]. Kudo et al. reported that human LAD2 mast cells produced *TNF-α* upon infection. Human and mouse mast cells are known to express *TLR4*. *TLR4* present on mast cell surfaces could recognize LPS from Gram-negative bacteria and produced *TNF-α* as a response [14,28]. Most of the previous reports were based on LPS derived from *E. coli*; therefore, we investigated the immune responses of mast cells to LOS derived from *A. baumannii*, via TLR4. In the present study, LAD2 cells showed constitutive *TLR4* expressions after stimulation with *A. baumannii* LOS derived from ATCC 19606 and MDRA T14 (Figure 1E). The expression of *TLR4* remained constant when treated with 5 to 100 ng/mL of LOS derived from *A. baumannii*, but increased at a concentration of 1000 ng/mL (Figure 1F).

The human LAD2 mast cells released TNF-α and IL-8 in a dose-dependent manner upon stimulation with *A. baumannii* LOS (Figure 2A,B). The pathogenicity of *A. baumannii* is often compared to that of *P. aeruginosa,* which was believed to have the ability to cause serious nosocomial infections [8], biofilm formation, and development of drug resistance [29,30]. We observed significant levels of TNF-α produced by stimulation with ATCC 19606- and MDRA T14-derived LOS (Figure 2, Figure S3). Kudo et al. reported that LAD2 cells showed increased TNF-α production when treated with LPS derived from *E. coli* [14]. Our results suggested that TNF-α production by the LAD2 cells was induced by LOS stimulation.

We previously reported that LAD2 cells infected with *A. baumannii* with a multiplicity of infection (MOI) 50 released about 70 pg/mL TNF-α and 60 pg/mL IL-8, and both of these pro-inflammatory mediators in culture medium activated neutrophil transmigration [15]. However, in this study, the levels of these two pro-inflammatory mediators after LOS stimulation were lower than those from *A*. *baumannii*-infected LAD2 cells in a previous study. TNF-α production from LAD2 cells stimulated with 10 ng/mL ATCC 19606, and MDRA T14 LOS was 9.8 pg/mL and 3.8 pg/mL, whereas IL-8 production was 16 pg/mL and 17 pg/mL, respectively. Stimulation with 100 ng/mL or 1 µg/mL of MDRA T14 LOS induced higher IL-8 production in LAD2 cells than in cells stimulated with LOS from ATCC 19606. These results indicated that LOS from *A. baumannii*, in particular, contributed to the production of IL-8 from LAD2 cells.

It has been reported that mouse bone marrow-derived mast cells release pro-inflammatory mediators upon *E. coli* LPS stimulation; this promotes mouse neutrophil effector functions (migration, phagocytosis, and generation of reactive oxygen species) [31]. TNF-α and IL-8 play crucial roles in the activation and recruitment of neutrophils to inflammatory sites [11,31,32]. Using an in vitro trans-well co-culture model, we showed that the migration of human peripheral neutrophils was dependent on TNF-α or IL-8 (Figure 3). A higher number of transmigrated neutrophils in the group in which LAD2 cells were stimulated with MDRA T14-LOS, as compared to other groups, reflected the critical role of IL-8 in the inflammatory response.

Bacterial LPS/LOS are the major components of the outer surface of Gram-negative bacteria. LPS/LOS are often of interest for their immunomodulatory properties and are one of the most potent stimulators of the host’s innate immune system. LPS/LOS recognition is essential for the host organism to eradicate invading bacterial pathogens. Recently, several studies using ATCC strains or clinical isolates with multi-drug resistance have reported that mutations or deletions of genes (e.g., *LpxA, LpxC, LpxD,* or *LptD*) involved in LOS biosynthesis or transport are strongly associated with the pathogenicity of *A. baumannii* [33,34,35,36]. Ubagai et al. reported that MDRA T14 LOS stimulation resulted in a higher expression of pro-inflammatory cytokines in neutrophils than stimulation with ATCC 19606 or other bacterial LOS [37]. We preliminary observed that LOS derived from MDRA T14 showed slightly lower molecules in intermediate saccharide chains compared to ATCC 19606. (Figure S1). It might reflect the difference in levels of acylation or phosphorylation of their lipid A. However, further research on the structural analysis of LOS derived from MDRA T14 is required and can unravel the exact mechanism of the LOS-mediated immune responses.

## 4. Conclusions

The present study is the first report demonstrating the effect of stimulation of LOS derived from *A. baumannii* on innate immune responses of mast cells. *A. baumannii* LOS stimulation strongly induced pro-inflammatory gene expression in mast cells. The findings suggested that *A. baumannii* LOS might determine the virulence and pathogenicity in inflammatory responses via mast cells. In addition, higher levels of IL-8 production indicated its role in recruiting neutrophils in response to bacterial invasion in the presence of LOS stimuli.

## 5. Materials and Methods

### 5.1. LAD2 Cell Culture

The human mast cell line LAD2 was a kind gift from Dr. Kirshenbaum [38] and was grown in StemPro-34 serum-free medium (Life Technologies, Grand Island, NY, USA) supplemented with 2 mM L-glutamine, 100 IU/mL penicillin, 100 mg/mL streptomycin (Life Technologies), and 100 ng/mL recombinant human stem cell factor [39]. Maturation of LAD2 cells was confirmed by monitoring their morphological features and the presence of metachromatic granules stained by toluidine blue.

### 5.2. LOS Preparation

For isolation of lipooligosaccharides (LOS), Two bacterial strains—*A. baumannii* ATCC 19606 and multidrug-resistant strains *A. baumannii* (MDRA T14)—were used. MDRA T14 were isolated from clinical isolates from the Teikyo University Hospital (Tokyo, Japan). LOS from ATCC 19606 and MDRA T14 were obtained according to phenol extraction [40]. Briefly, bacteria were cultured in Luria–Bertani broth at 37 °C overnight and harvested. After harvesting, the bacteria were washed with distilled water. The washed bacteria were treated successively with ethanol, acetone, and twice with ether and lyophilized. Further purification was processed on consignment by a chemical-industrial company (Wako Pure Chemical Industries, Ltd., Osaka, Japan). The lyophilized bacterial masses were digested with lysozyme. Nucleic acids were degraded by treatment with DNase and RNase. Bacteria proteins were digested by incubation with proteinase K. The digested cells were extracted twice with 45% phenol/water at 68 °C, and the separated layers were dialyzed against deionized water [40]. Pure LOS preparations were obtained by ultracentrifugation twice. Purified LOS contained <1% protein by Bradford method, and the endotoxin levels of the LOS obtained from different strains were >500,000 EU (endotoxin units)/mg by LAL Endotoxin assay (ToxiSensor Chromogenic LAL Endotoxin Assay Kit; GenScript, Piscataway, NJ, USA).

### 5.3. Mast Cells Stimulation with LOS

LAD2 cells (2 × 10^6^ cells mL^−1^ per well) were seeded in a non-treated 24-well plate (Iwaki, Asahi Glass Co., Ltd., Tokyo, Japan) supplemented with StemPro-34 medium. Subsequently, LOS (10 ng/mL to 1 µg/mL) was added to the wells, and the plates were incubated at 37 °C for 4 h under 5% CO_2_. The supernatants from mast cells were obtained and assayed using the Cytometric Bead Array Human Inflammation Kit (CBA, BD Biosciences, San Diego, CA, USA) following the manufacturer’s instructions.

### 5.4. RNA Preparation

Following 1 h stimulation with LOS at different concentrations (10 ng/mL to 1 µg/mL), the total RNA from cells was extracted using the RNeasy Mini Kit (QIAGEN, Hilden, Germany), following the manufacturer’s instructions. The quantity and quality of the total RNA samples were determined using an Agilent 2100 Bioanalyzer (Agilent Technologies, Waldbronn, Germany).

### 5.5. Complimentary DNA Synthesis

Total RNA was reverse-transcribed to cDNA using the SuperScript VILO cDNA Synthesis Kit for RT-PCR (Life Technologies) following the manufacturer’s instructions. Briefly, the reaction was carried out in a 20 µL reaction volume containing 1 µg RNA, 2 µL SuperScript Enzyme mix, 4 µL 5× VILO Reaction Mix, and DEPC-treated water. The components were combined in a tube on ice and mixed gently. Subsequently, the samples were incubated at 25 °C for 10 min, followed by incubation at 42 °C for 120 min, and finally, the reactions were terminated at 85 °C for 5 min.

### 5.6. Quantitative Real-Time PCR (qRT-PCR) Analysis

Gene-expression levels of *TNF-α* (GenBank Accession No. NM_000594), *IL-8* (GenBank Accession No. NM_000584), *CCL4* (MIP-1β) (Genbank Accession No. NM_002984), *LTC4S* (Genbank Accession No. NM_145867), and *TLR4* (Genbank Accession No. NP_003257) in LAD2 cells were quantified using the ABI StepOne Real-Time PCR System (Applied Biosystems, Foster City, CA, USA). cDNAs were amplified with SYBR Green using the Platinum SYBR Green qPCR SuperMix UDG (Invitrogen, Carlsbad, CA, USA). Quantitative PCR (qPCR) was performed for *TNF-α, IL-8*, *CCL4,* and the reference gene, *β-Actin* (GenBank Accession No. NM_01101). The primer sets (Table 1) were designed using Primer3 Plus (http://www.bioinformatics.nl/cgi-bin/primer3plus/primer3plus.cgi (accessed on 1 January 2021)). The PCR conditions for amplification of cDNA were as follows: 50 °C for 2 min; 95 °C for 10 min followed by 40 cycles of 95 °C for 15 s, 59 °C for 30 s, 72 °C for 30 s, and one cycle at 60 °C for 1 min. The expression levels of the target genes in LAD2 cells were estimated after incubation with LPS for 1, 2, 4, and 6 h and were normalized to β-actin gene-expression levels. Eventually, the fold changes in expression of cytokine mRNA between the bacterial infected cells and control cells were determined using the Sequence Detection systems software (Applied Biosystems) [15,36]. The real-time PCR result with at least two-fold expression change was considered significant [41].

### 5.7. Cytokine Assay

The concentration of six inflammatory cytokines (IL-1β, IL-6, IL-8, IL-10, IL-12p70, and TNF-α) in the culture supernatants of LAD2 cells incubated with LOS for 4 h was determined using the Cytometric Bead Array Human Inflammation Kit [15,42] according to the manufacturer’s instructions. Samples were analyzed using a BD FACS Canto II flow cytometer (BD Biosciences). Acquisition of events and analysis of results was performed using FCAP Array Software Version 3.0 (BD Biosciences).

### 5.8. Neutrophil Preparation

Human neutrophils were isolated from the peripheral blood of healthy volunteers, as previously described [15]. Briefly, heparinized whole blood was mixed with 4.5% dextran solution and allowed to stand for 40 min at 20 °C. The neutrophil-rich plasma was then subjected to density-gradient centrifugation at 400× *g* for 30 min, using a Lymphosepar I (Immuno-Biological Laboratories, Tokyo, Japan). To lyse erythrocytes, hypotonic (0.2%) saline was used, and the osmolality was restored with hypertonic (1.6%) saline. The purity of the neutrophils was >95%, as assessed by Diff-Quik staining.

All participants were informed about the purpose of this research project, and informed, written consent was obtained before the study. The protocol was approved by the Ethical Review Committee at Teikyo University School of Medicine (No. 07-104).

### 5.9. Cell-Migration Assay

In vitro cell-migration assays were performed in a Boyden-chamber system (Falcon 3504 24-well culture plate; Falcon 3492 cell culture insert with a polyethylene terephthalate (PET) membrane [3.0 µm pore size]; BD Biosciences), as described previously [15,43,44]. Neutrophil suspension in StemPro-34 medium (1 × 10^5^ cells) was seeded in the upper chamber of the cell-culture insert. The lower chamber was filled with 0.6 mL of LAD2-culture supernatant, with or without LOS. The chambers were incubated at 37 °C for 1 h under a 5% CO_2_ atmosphere. The neutrophils remaining on the upper surface of the membrane were wiped off with a cotton swab. Then the cells that had migrated to the bottom surface of the membrane were stained with crystal violet (5 mg/mL) in 20% methanol for 5 min and counted under a microscope (Olympus BX53, Olympus, Tokyo, Japan).

## Figures and Tables

**Table 1:** Amplicon lengths and sequences of oligonucleotide primers used in quantitative real-time PCR.

Gene	Sequence	Amplicon Length (bp)
*TNF-a*	F: 5′-AGACCAAGGTCAACCTCCT-3′ R: 5′-AAAGTAGACCTGCCCAGAC-3′	194
*IL-8*	F: 5′-ATCCACAAGTCCTTGTTCCA-3′ R: 5′-AAGTGCTTCCACATGTCCTC-3′	113
*CCL4*	F: 5′-GCCTGCTGCTTTTCTTACAC-3′ R: 5′-CTTGCTTCTTTTGGTTTGGA-3′	117
*LTC4S*	F: 5′-AGGTGGGCTGGTTCCTATCTA-3′ R: 5′-CCCATGGCTATCCTACCATTT-3′	220
*TLR4*	F: 5′-ATTTCAGCTCTGCCTTCACTA-3′ R: 5′-CTTCTGCAGGACAATGAAGAT-3′	212
*β-actin*	F: 5′-TTAAGGAGAAGCTGTGCTACG-3′ R: 5′-TTGAAGGTAGTTTCGTGGATG-3′	205

## Supplementary Materials

The following are available online at https://www.mdpi.com/2076-0817/10/3/290/s1, Figure S1: SDS-PAGE and silver staining analysis of *A. baumannii* lipooligosaccharides. Figure S2: *A. baumannii* infection induced mRNA expression of the *CCL4*. Figure S3: Pro-inflammatory cytokines production of LAD2 cells stimulated with LPS derived from *Pseudomonas aeruginosa,* PAO1 and Multi-drug resistant clinical isolate (MDRP).
