# Supplementary material for: Acinetobacter baumannii LOS Regulate the Expression of Inflammatory Cytokine Genes and Proteins in Human Mast Cells
Source: Pathogens. 2021 Mar 3;10(3):290. doi: 10.3390/pathogens10030290 (PMC7998227; doi:10.3390/pathogens10030290)
Supplement: Supplementary file 1 [file pathogens-10-00290-s001.pdf]

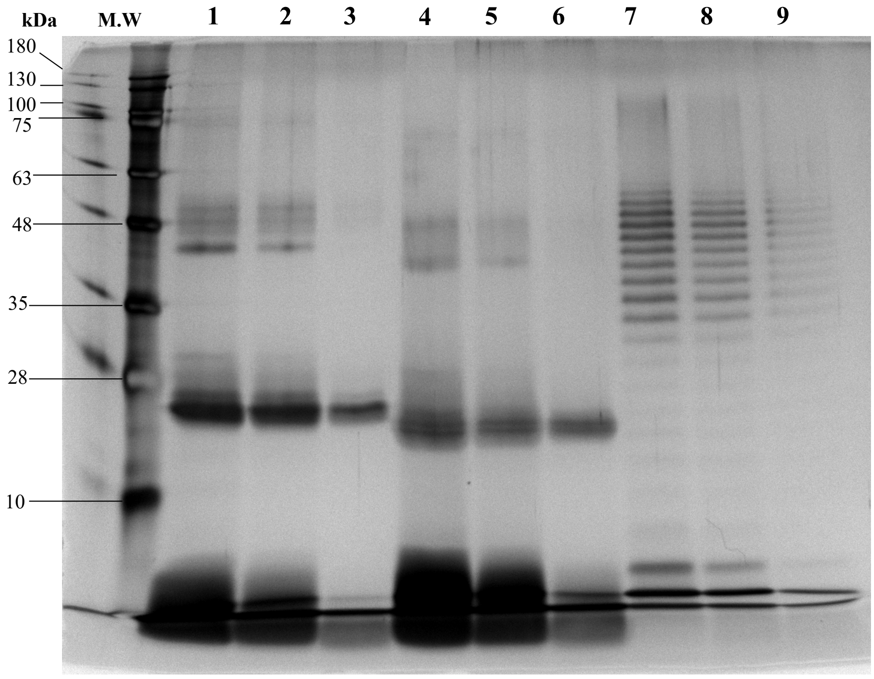

Figure S1. SDS-PAGE and silver staining analysis of *A. baumannii* lipooligosaccharides. Lane 1, ATCC 19606, 10 µg; lane 2, 5 µg; lane 3, 1 µg, lane 4, MDRA T14, 10 µg; lane 5, 5 µg; lane 6, 1 µg, lane 7, *E. coli* O111: B4 (LPS), 10 µg; lane 8, 5 µg; lane 9, 1 µg. Each LOS sample was treated for 5 min at 100 ˚C in 0.05 M Tris hydrochloride buffer (pH 6.8), 2 % SDS and 0.01 % bromophenol blue, and fractionated on an SDS-polyacrylamide gel containing 4 % and 14 % acrylamide with 4M urea in the stacking and separating gels, respectively. LOS preparations were stained by the conventional silver staining method according to manufacturer's instructions (Cosmo Bio, Tokyo, Japan).

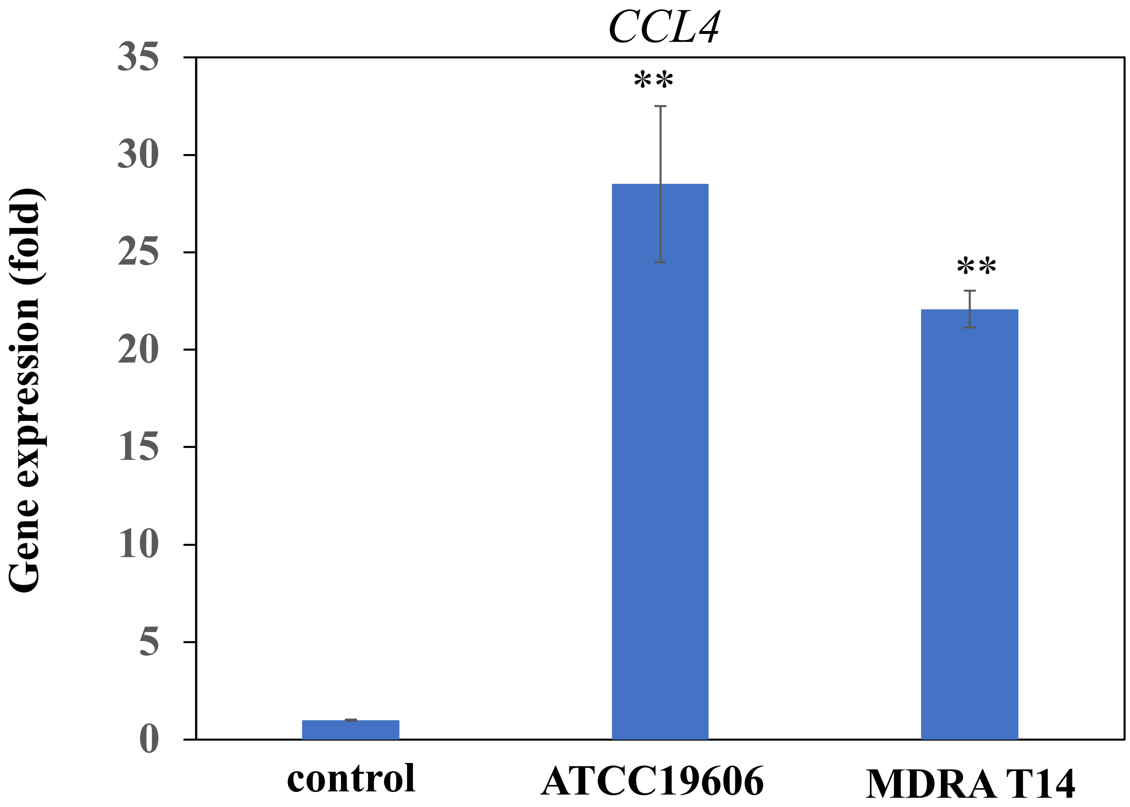

Figure S2. *A. baumannii* infection induced mRNA expression of the *CCL4*. Shown is the mRNA-expression ratio compared with that for LAD2 cells cultured medium (control) after infection with *A. baumannii* (ATCC 19606, MDRA T14) at an MOI of 50 for 4h. Fold-changes are presented. $^{**}P < 0.01$, compared with LAD2 control. Error bars represent the standard error. The data shown are representative of at least 3 independent experiments.

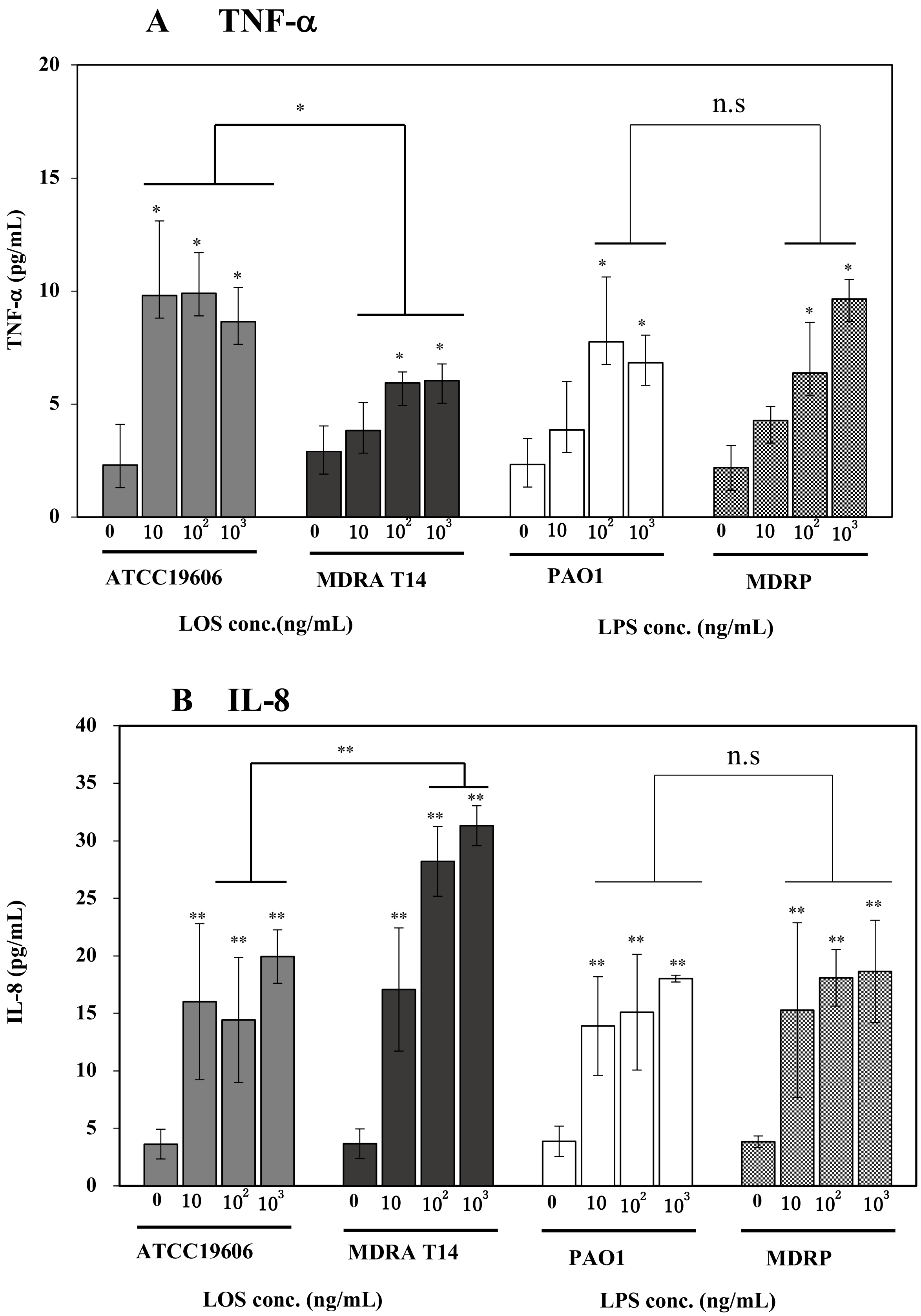

Figure S3. Pro-inflammatory cytokines production of LAD2 cells stimulated with LPS derived from *Pseudomonas aeruginosa*, PAO1 and Multi-drug resistant clinical isolate (MDRP). (A) TNF-a and (B) IL-8. LPS from PAO1 was purchased from Sigma-aldrich. LPS from MDRP was purified by the same protocol of LOS of *A. baumannii* in this study. LAD2 cells (2×10^6 cells mL^{-1} per well) were seeded in a non-treated 24-well plate supplemented with StemPro-34 medium. Subsequently, LOS from *A. baumannii* ATCC 19606 and MDRA T14 (10 ng/mL to 1 μg/mL) or LPS from *P. aeruginosa* PAO1 and MDRP (10 ng/mL to 1 μg/mL) were added to the wells and the plates were incubated at 37°C for 4 h under a 5% CO_2. The supernatants from mast cells were obtained and assayed using the Cytometric Bead Array Human Inflammation Kit (CBA, BD Biosciences, San Diego, CA, USA) following the manufacturer's instructions.
